# A novel application of laser speckle imaging technique for prediction of hypoxic stress of apples

**DOI:** 10.1186/s13007-024-01271-7

**Published:** 2024-09-28

**Authors:** Piotr Mariusz Pieczywek, Artur Nosalewicz, Artur Zdunek

**Affiliations:** Institute of Agrophysics, Polish Academy of Sciences, Doświadczalna 4, 20-290 Lublin, Poland

**Keywords:** Laser speckle imaging, Low oxygen atmosphere, Stress, Apple, Modelling, Respiration rate, Chlorophyll fluorescence

## Abstract

**Background:**

Fruit storage methods such as dynamic controlled atmosphere (DCA) technology enable adjusting the level of oxygen in the storage room, according to the physiological state of the product to slow down the ripening process. However, the successful application of DCA requires precise and reliable sensors of the oxidative stress of the fruit. In this study, respiration rate and chlorophyll fluorescence (CF) signals were evaluated after introducing a novel predictors of apples' hypoxic stress based on laser speckle imaging technique (LSI).

**Results:**

Both chlorophyll fluorescence and LSI signals were equally good for stress detection in principle. However, in an application with automatic detection based on machine learning models, the LSI signal proved to be superior, due to its stability and measurement repeatability. Moreover, the shortcomings of the CF signal appear to be its inability to indicate oxygen stress in tissues with low chlorophyll content but this does not apply to LSI. A comparison of different LSI signal processing methods showed that method based on the dynamics of changes in image content was better indicators of stress than methods based on measurements of changes in pixel brightness (inertia moment or laser speckle contrast analysis). Data obtained using the near-infrared laser provided better prediction capabilities, compared to the laser with red light.

**Conclusions:**

The study showed that the signal from the scattered laser light phenomenon is a good predictor for the oxidative stress of apples. Results showed that effective prediction using LSI was possible and did not require additional signals. The proposed method has great potential as an alternative indicator of fruit oxidative stress, which can be applied in modern storage systems with a dynamically controlled atmosphere.

**Supplementary Information:**

The online version contains supplementary material available at 10.1186/s13007-024-01271-7.

## Background

Apples (*Malus x domestica* Borkh.) are usually stored in cold rooms under a controlled atmosphere (CA) in which O_2_ level is reduced to maintain fruit quality [1, 2]. Studies show that storage in oxygen-reduced atmosphere conditions is beneficial in maintaining adequate levels of product firmness, vitamin, organic acid and sugar content and preventing or reducing the risk of storage damage, especially surface scald [3–8]. The optimal level of O_2_ in CA was determined by trial and error, but remain above the anaerobic compensation point (APC)—the O_2_ level at which the CO_2_ production is minimal [9]. Below the ACP the risk of severe quality losses increases due to the anaerobic metabolism (fermentation) that starts to dominate the metabolic activity of fruit [10–12]. However, determining the minimum O_2_ level at which anaerobic stress occurs depends on many factors, such as the variety of fruit, the degree of ripeness, and the region of harvest determining growth conditions [13].

To address this problem a dynamic controlled atmosphere technology has been developed to adjust the oxygen level in the storage atmosphere, according to the physiological state of the product, which is closely monitored using low-oxygen sensors [2, 14]. With the detection of anaerobic stress, the composition of the storage atmosphere is adjusted to maintain the O_2_ level as close as possible above the acceptable low oxygen level (LOL) value of the product. The two most prominent types of dynamic controlled atmosphere systems use measurements of chlorophyll-fluorescence (DCA-CF) and respiratory quotient (DCA-RQ) as indicators of low-oxygen levels [1–3, 5, 15–17].

The DCA-CF method is based on the observation that the minimum chlorophyll fluorescence of a dark-adapted sample of plants is sensitive to low-oxygen stress [3, 18]. The characteristic peak of the CF signal is observed when the O_2_ levels reach the LOL of the product. The DCA-CF method is primarily used in apple and pear storage [5, 7, 15, 19]. Despite its many advantages, the method also has some drawbacks. The content of chlorophyll, which is a biomarker of stress in DCA-CF systems, decreases during storage. It depends on the variety of fruit and can change due to environmental factors occurring during fruit ripening (e.g., light intensity). Chlorophyll fluorescence values are also susceptible to storage temperature and overall cell metabolic activity [17].

In the DCA-RQ method, the ratio of CO_2_ produced to O_2_ consumed by fruit serves as a biomarker used to detect anaerobic stress [2]. In an apple, as atmospheric oxygen levels decrease, oxygen uptake also decreases, but the RQ ratio remains relatively constant at around 1.0. As the ACP is reached, the RQ ratio increases significantly, due to an increase in CO_2_ production from fermentation processes. The O_2_ concentration corresponding to ACP is often close to the LOL value in apples [20]. However, determining the level of physiological stress based on the content of fermentative metabolites is complicated, because their production can occur above the LOL value [21]. Oxygen, CO_2_ and glycolysis products can be produced or consumed by many intracellular processes, making it difficult to interpret their concentration in the context of respiration and fermentation processes.

In this study a novel sensor of apples hypoxic stress based on laser speckle imaging (LSI) technique was introduced. Laser speckle imaging is a nondestructive and contactless method based on analysis of speckle pattern resulting from coherent light scattering on surface of biological objects [22]. The method is non-invasive and relatively simple to apply, hence it has so far found wide application in medicine and biological sciences. Spatio-temporal feature analysis of laser speckle images has been used for simultaneous quantification of skin thickness and microcirculatory flow rate [23]. It has also been demonstrated that laser speckle imaging enables the quantitative estimation of the inhibition zone of antibiotics regardless of bacterial species and types of antibiotics [24]. Temporal evolution of speckle patterns was also used as an non-invasive and low-cost technique for sizing dielectric microparticles [25]. One of the most frequently cited applications of this technique is blood flow imaging, where dynamic light scattering theory was proved to be effective, non-scanning and wide-field method [26]. The applications of the laser speckle imaging in agriculture include the determination of the quality and the degree of maturation of fruits and vegetables [23–27], detection of fungal infection [28] or monitoring of the ripening progress [29, 30]. Laser imaging supported by Artificial Neural Networks allowed for early detection of fungal infection in citrus using with accuracy of 94.0% reported for test set [31]. High correlation between biospeckle activity and germination trait was obtained, allowing for automatic, non-destructive, inexpensive assessment of seed priming treatments [32]. The dynamics of speckle patter were shown to be sensitive to the surface temperature [33] and the chlorophyll content of apple fruit [34]. As is was shown in recent work [35], the characteristics of the speckle signal are strongly related to the rate of respiration of the fruit. Anaerobic stress is the result of a change in the mode of respiration of the fruit from aerobic to anaerobic. Hence, it was hypothesized that speckle imaging could be an effective tool for detecting this phenomenon.

This study aimed to assess the applicability of laser speckle imaging as a predictor of anaerobic stress conditions in apples during storage in low oxygen conditions. The potential detection capabilities of laser speckle imaging signals were evaluated by measuring respiration rate and chlorophyll fluorescence signals. Finally, machine learning algorithms were employed to estimate the discriminatory power of laser speckle imaging in the automatic detection of hypoxia stress of apples. This is the first study presenting successful application of laser speckle imaging techniques to detect anaerobic stress in apple.

## Results

**Fig. 1:**
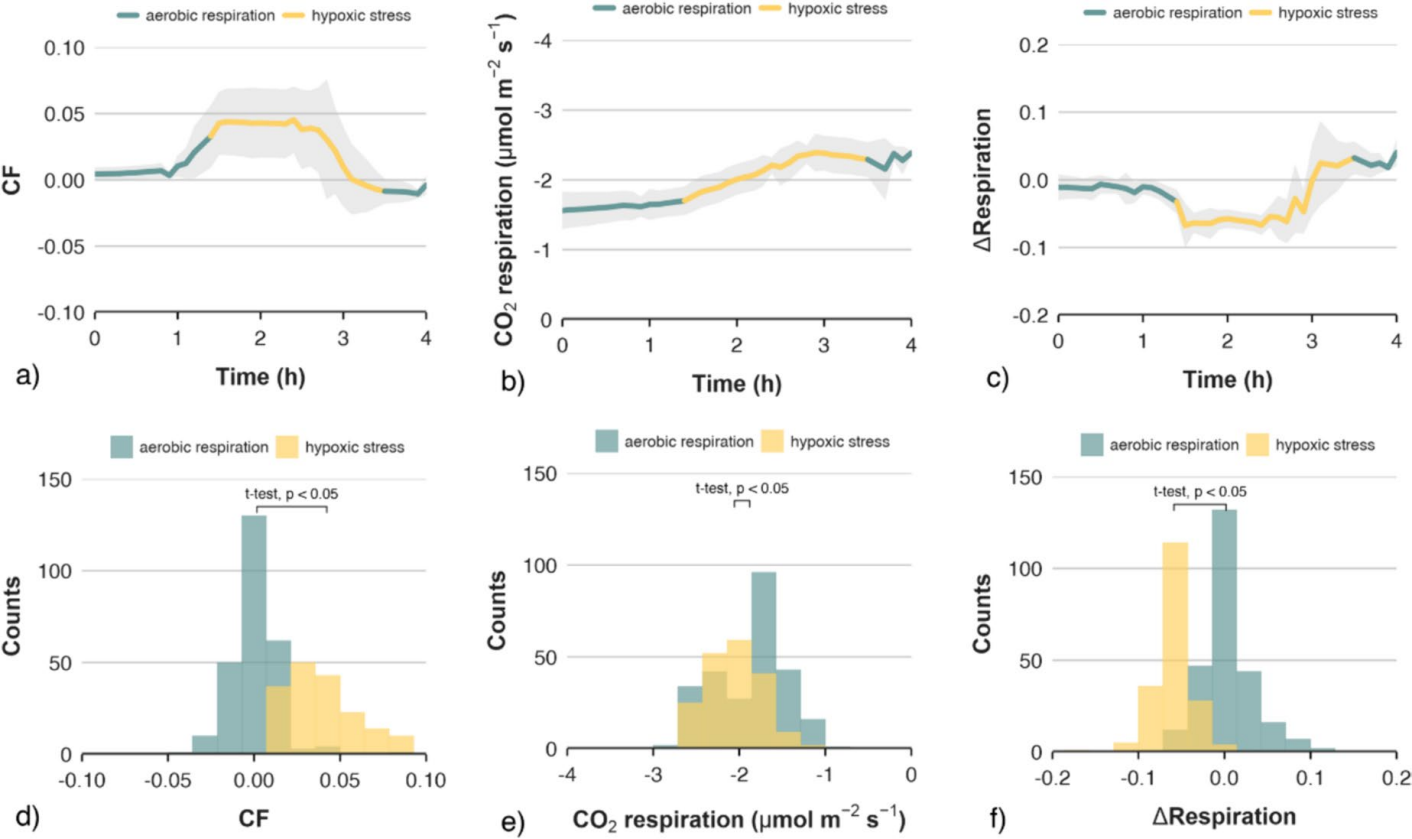
Averaged time series of chlorophyll fluorescence (**a**), CO_2_ respiration rate (**b**) and respiration rate first derivative (**c**) signals. Signals start one hour after reaching zero-oxygen atmosphere, showing 2.5 h under stress conditions, 0.5 h of transition phase and finally 1 h under normal conditions. The grey bands indicate the range of one standard deviation from the mean. The histograms show the distribution of chlorophyll fluorescence (**d**), CO_2_ respiration rate (**e**) and respiration rate first derivative (**f**) signal values with respect to two categories of the physiological state of the fruit

Apples stored in low-oxygen conditions showed typical time profiles of minimum chlorophyll fluorescence and CO_2_ respiration levels. Elevated CF signal (Fig. 1a), associated with hypoxic stress, was observed for almost all investigated apples. An increase in CF was followed by an increased CO_2_ respiration rate (Fig. 1b). The first noticeable changes in the case of CF indicators were, on average, observed 2.1 h after the removal of oxygen from the climate chamber. In this study, elevated levels of CF were associated with a monotonic increase in CO_2_ respiration for apples (Fig. 1b). The changing rate of respiration was reflected by the first derivative of the signal (Fig. 1c), which showed clear differences in the respiration dynamics of the fruit associated with aerobic respiration and hypoxic stress. The CO_2_ respiration did not drop to its initial level within one hour of restoring the normal ambient atmosphere (Fig. 1b). Despite the relatively rapid rate of change of atmosphere, the measurement time after return to normal atmosphere may be insufficient to capture the post-stress response of the fruit.

**Fig. 2:**
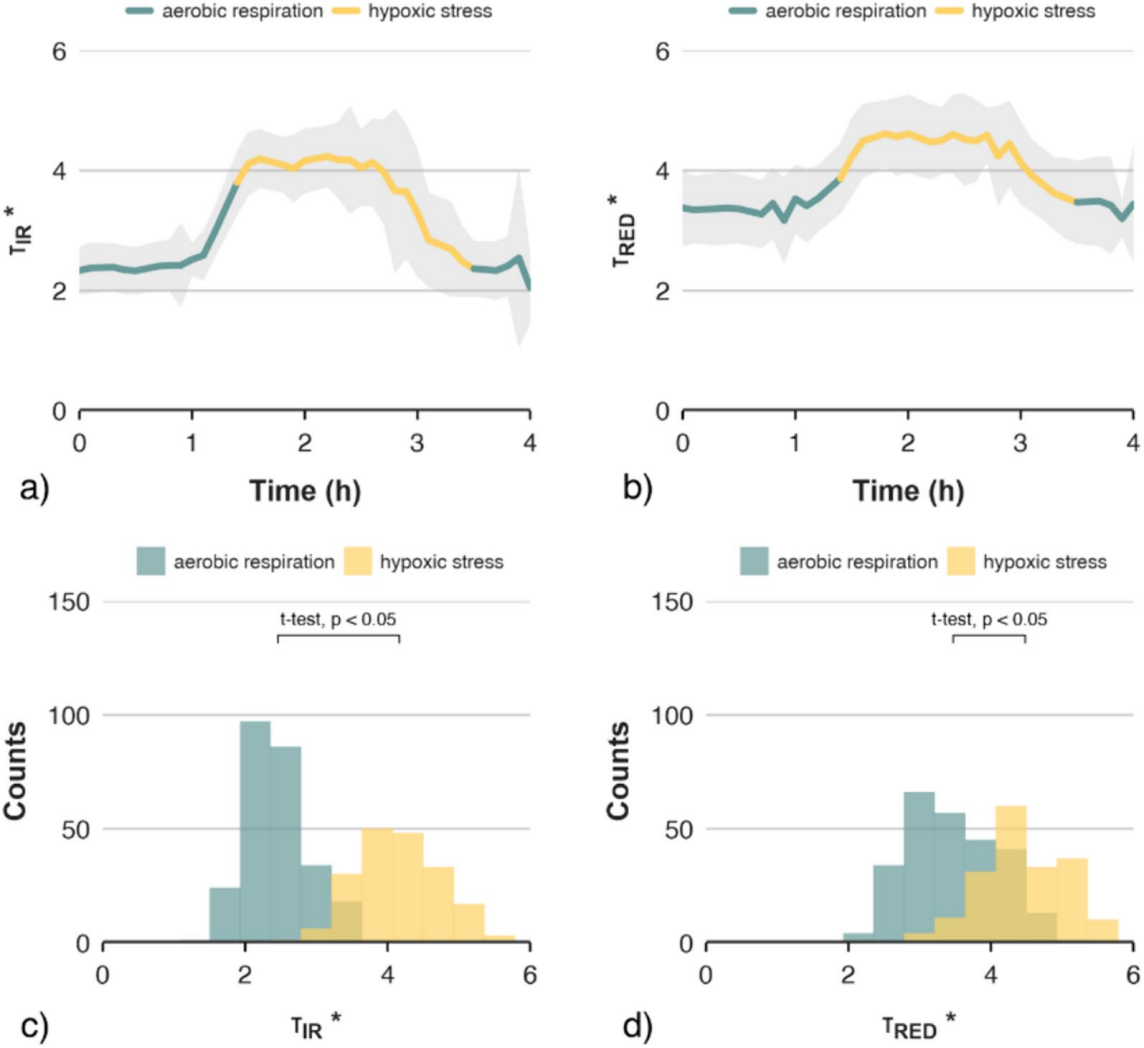
Averaged time series of speckle pattern relaxation time coefficients τIR* (**a**) and τRED* (**b**) for infra-red and red lasers, respectively. Signals start one hour after reaching zero-oxygen atmosphere, showing 2.5 h under stress conditions, 0.5 h of transition phase and finally 1 h under normal conditions. The grey bands indicate the range of one standard deviation from the mean. The histograms show the distribution of speckle pattern relaxation time coefficients τIR* (**c**) and τRED* (**d**) with respect to two categories of the physiological state of the fruit

**Fig. 3:**
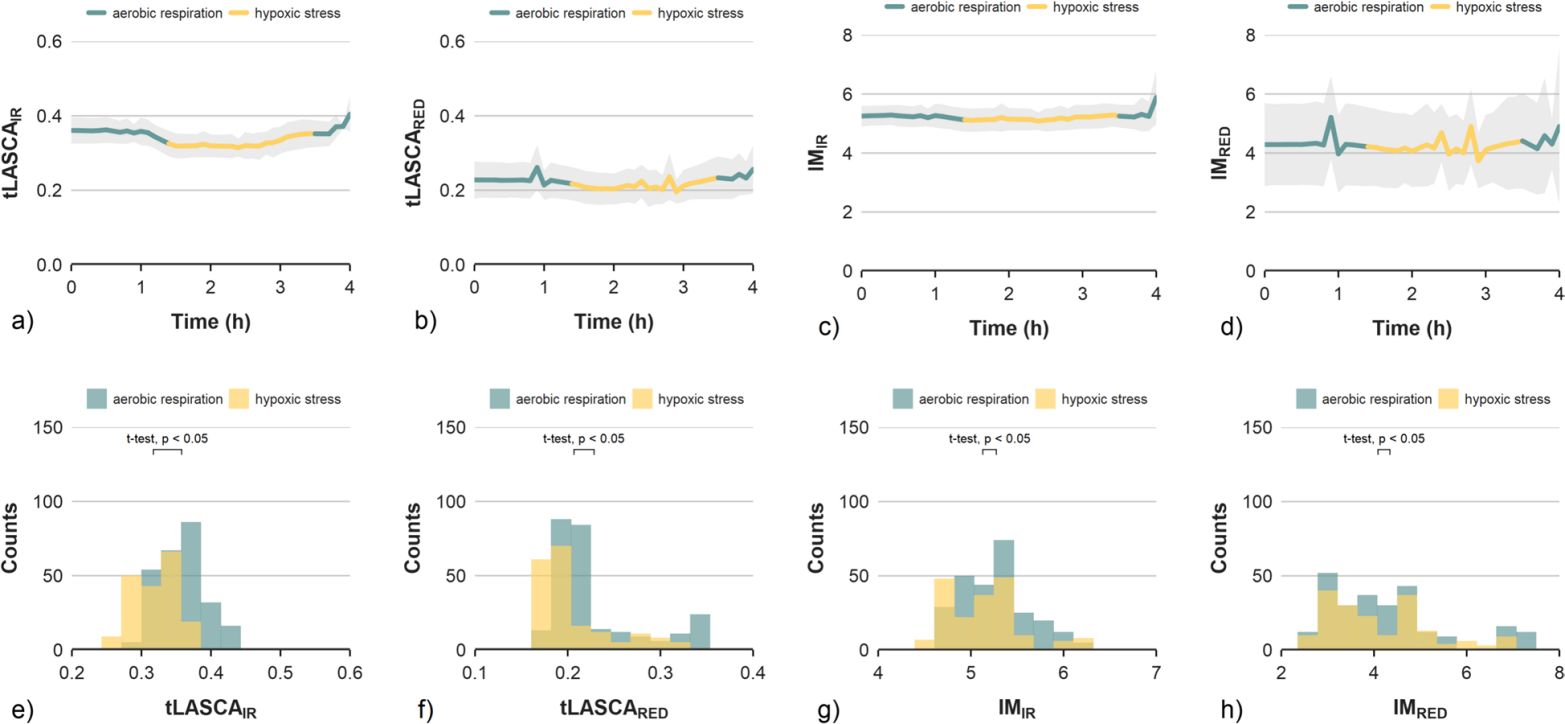
Averaged time series of speckle pattern fluctuations coefficients: tLASCA for infra-red (**a**) and red lasers (**b**) and IM for infra-red (**c**) and red lasers (**d**), respectively. Signals start 1 h after reaching zero-oxygen atmosphere, showing last 2.5 h under stress conditions, 0.5 h of transition phase and finally 1 h under normal conditions. The grey bands indicate the range of one standard deviation from the mean. The histograms show the distribution of speckle pattern fluctuation coefficients with respect to two categories of the physiological state of the fruit for tLASCA for infra-red (**e**) and red lasers (**f**) and IM for infra-red (**g**) and red lasers (**h**)

Like the CF signal, both laser speckle relaxation time coefficients (τ_RED_* and τ_IR_*) followed the pattern of rising to a constant level and decreasing after returning to the normal atmosphere (Fig. 2). Slight differences in detection moment between applied methods were noticed, i.e., 2.04, 2.10 and 2.17 h for laser speckles, CF signal and respiration rate, respectively. However, differences were statistically insignificant and small enough to be attributed to misalignment in synchronisation between measuring devices (less than eight minutes which was within the interval between measurements). The laser speckle activity calculated using methods based on measurements of changes in pixel brightness (IM or LASCA) was considerably lower, compared to τ_RED_* and τ_IR_*. Although signal fluctuations corresponding to the onset of oxidative stress were evident, the magnitude of the differences was small compared to the other methods. Both IM and LASCA were not able to clearly identify the states associated with aerobic respiration and hypoxic stress (Fig. 3). The low discriminatory power of the signals was independent of the type of laser used. This was confirmed by the distribution of values corresponding to the two physiological states of the fruit, which largely overlapped (Fig. 3e–h), in contrast to the coefficients based on CF, CO_2_ consumption (Fig. 1) and laser speckle relaxation time coefficients (τ_RED_* and τ_IR_*, see Fig. 2).

Table S1 included in the supplementary materials provides data on mass, firmness, soluble solid content (SSC) volume and surface area for each of the apples tested. Fruit mass ranged over a relatively wide range from 145.5 to 202.4 g, with an average value of 163.92 ± 12.87 g (standard consumption apples). Much smaller variations in values were observed for firmness and SSC. The mean values and standard deviations were 41.15 ± 3.32 and 12.67 ± 0.92 for firmness and SSC, respectively. These were typical values for ripe fruit after storage. The area of the smallest fruit compared to the largest differed by 27%. However, this was due to the presence of outlier fruit in the sample. For the remaining fruits, the average area was equal to 154.3 cm^2^ and deviations from this value did not exceed 4%.

## Discussion

### Response of apples to a low-oxygen atmosphere

A sudden reversible peak in the CF profile in response to low-oxygen atmosphere had already been observed and well documented in previous studies [5, 15, 19, 36]. Changes in CF have been considered a highly effective indicator of acceptably low oxygen levels for a given product [16]. However, one of the disadvantages of this approach is the sensitivity to different levels of chlorophyll, which vary with the maturity of the fruit [37]. In this study, the raw time series of CF signals differed significantly in initial values as well as values obtained during stress, depending on the fruit and even the location of the fruit from which data was collected. Even after normalisation, the CF signals associated with hypoxic stress showed considerable deviation from the mean value (Fig. 1a). In some individual cases, an increase in CF was not observed at all, while other methods showed characteristic signal changes. The overall respiration rate of apples is known to decrease with the storage O_2_ concentration [20, 38, 39]. This decrease is observed up to a critical oxygen concentration at which the anaerobic compensation point (ACP) is reached. Below the ACP fruit respiration changes from primarily aerobic to anaerobic (fermentation), resulting in increased CO_2_ production.

As mentioned, both τ_IR_* and τ_RED_* signals provided a clear distinction between aerobic respiration and hypoxic stress conditions (Fig. 2), showing trends comparable to the CF signal. This is the first study in which this effect was observed for the signal from the laser speckle imaging technique. Unlike the CF signal, τ_IR_* did not require normalisation. The τ_IR_* signal values were reproducible and comparable between fruits, showing a much smaller deviation from the mean value, compared to CF. A much greater deviation of values was observed for τ_RED_*, compared to τ_IR_* signal. This effect could be caused by different concentrations of pigments in the skin of the fruit, and higher susceptibility of red light to the presence of various chemical compounds in the apples, compared to infrared light [22, 40]. A study on post-harvest monitoring of tomato fruit showed a higher correlation of speckle signal with chlorophyll content for red laser, compared to infra-red laser light [41].

### Discriminatory power of respiration rate, chlorophyll fluorescence and laser speckle imaging signals

Before modelling, an analysis of predictor variables was carried out to evaluate the discriminatory power of signals collected from different types of indicators of oxidative stress. Table 1 shows the correlations between all the possible pairs of the measured parameters and the categorical variable describing two physiological states of apples—aerobic respiration and hypoxic stress. The discriminatory capabilities of the applied predictors were also assessed based on the histograms of values, for two categories of signals (Figs. 1d–f and 2c, d). The detection capabilities of oxidative stress of laser speckle imaging varied depending on the method of signal processing.

**Table 1:**
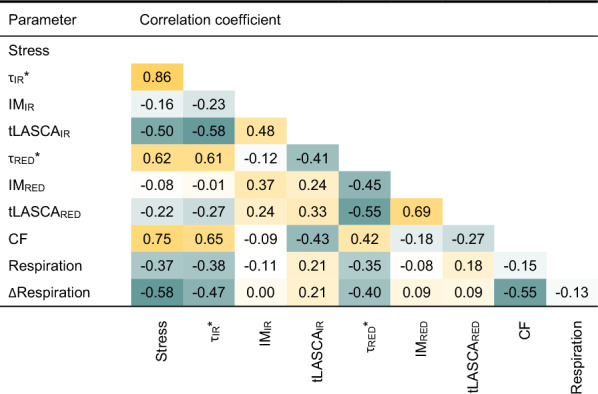
Correlation matrix of model variables for all 605 observations

Both τ_RED_* and τ_IR_* signals showed good discriminatory capabilities, with superior results for the latter. The τ_IR_* signal showed the highest correlation with stress (0.86, see Table 1), splitting observations into two significantly different groups (see Fig. 2c) with the smallest overlap between values from both categories, among all tested methods. Due to the higher variance of values, the τ_RED_* coefficient showed a considerably lower correlation with the stress conditions of the apples (0.62, see Table 1). Weaker discriminating ability was indicated by the distribution of τ_RED_* values, which showed a significant overlap between both groups, despite statistically significant differences.

All the other coefficients calculated using the laser speckle imaging signal (IM, tLASCA) had surprisingly poor discriminating abilities. Only the tLASCA_IR_ showed a significant correlation with stress categorical variables. The IM_IR_, IM_RED_, tLASCA_RED_ showed not only the lowest correlations among all tested predictors but also none of these coefficients was able to provide a sufficiently good division of observations into two significantly different groups (Fig. 3). There are several possible reasons for this observed discrepancy. Methods like laser speckle contrast analysis require fine-tuning of image acquisition parameters and, most importantly, the exposure time to catch up with the dynamics of the observed process [22, 42]. In this study, all laser speckle coefficients were calculated from video sequences recorded with the same acquisition settings. It is possible that the selected settings were not optimal for one of the LSI methods. Laser speckle contrast analysis and inertia moment are considered to be intensity-based coefficients. Both reflect changes in pixel intensity levels, expressed using different statistics (mean value, standard deviation etc.). In contrast, the speckle pattern relaxation time calculation is based on temporal changes in the content of recorded video frames, expressed by the correlation coefficient. It is possible that the content-based approach is better at capturing signal changes related to the stress phenomena. For instance, signals from IM_IR_, IM_RED_ and τ_RED_* and τ_IR_* were uncorrelated.

Considering the above, only two coefficients based on the laser speckle imagining method were used in further modelling, namely τ_RED_* and τ_IR_*. The second highest correlation (0.75, see Table 1) between signal values and stress was reported for the chlorophyll fluorescence method. The distribution of the CF values also showed good separation of the signal categories, with only slightly greater overlap, compared to τ_IR_*. Although the differences between the mean values of respiration rate for the two signal categories were statistically significant (Fig. 1e), respiration rate was considered potentially the weakest predictor of stress, due to the significant overlap between the groups and the resulting low correlation coefficient with stress (Table 1). Significant improvements in discriminatory power were obtained for the first derivative of the respiration rate signal (ΔRespiration), resulting in good group separation (Fig. 1f) and a higher correlation coefficient compared to the raw respiration rate signal.

### Automatic detection of hypoxia stress in apples

The three variants of predictive models that were built for each of the four tested signals (τ_RED_*, τ_IR_*, CF and ΔRespiration) differ in the set of predictors used for training and testing. The predictive capabilities of the models—with the relative importance of the predictors—are presented in Table 2. Additional predictors included the mass, firmness and soluble solids content of the apples. These were considered to be possible indirect indicators of fruit maturity, which is known to affect the response of apples to stress conditions [43, 44]. However, the applicability of models built with firmness and soluble solids content is limited, as both parameters require destructive analysis, which can be carried out after measurements of τ_RED_*, τ_IR_*, CF or ΔRespiration. Nevertheless, examining the impact of these coefficients allowed us to indirectly assess the predictive power of the base signals.

**Table 2:** Prediction performance of different models

	Parameter importance							Model performance			
Model variant	τ_IR_*	τ_RED_*	CF	ΔResp.	Mass	Firmness	SSC	Accuracy	Precision	Recall	**F1 score**
1	1.00							0.91 (0.95)	0.92 (0.96)	0.95 (0.97)	0.93 (0.96)
2		1.00						0.78 (0.86)	0.81 (0.87)	0.86 (0.93)	0.84 (0.90)
3			1.00					0.90 (0.94)	0.92 (0.94)	0.93 (0.97)	0.93 (0.95)
4				1.00				0.82 (0.88)	0.87 (0.90)	0.86 (0.91)	0.86 (0.90)
5	0.89				0.11			0.97 (0.99)	0.98 (0.99)	0.98 (0.99)	0.98 (0.99)
6		0.70			0.30			0.88 (0.95)	0.90 (0.94)	0.93 (0.98)	0.91 (0.96)
7			0.86		0.14			0.95 (0.99)	0.96 (0.99)	0.96 (0.99)	0.96 (0.99)
8				0.85	0.15			0.84 (0.94)	0.88 (0.95)	0.89 (0.96)	0.88 (0.95)
9	0.89				0.02	0.07	0.02	0.96 (0.99)	0.96 (0.99)	0.97 (0.99)	0.97 (0.99)
10		0.66			0.12	0.10	0.12	0.88 (0.97)	0.89 (0.96)	0.93 (0.97)	0.91 (0.97)
11			0.83		0.06	0.05	0.06	0.94 (0.99)	0.95 (0.99)	0.96 (0.99)	0.95 (0.99)
12				0.83	0.08	0.03	0.06	0.86 (0.96)	0.88 (0.96)	0.88 (0.97)	0.88 (0.97)

Rows correspond to variants of trained models. Columns in the “Parameter importance” section show gain values for individual parameters with respect to model variants (rows). Gain values in the column indicate which parameters were used to build the model. A parameter was not used if the value is not specified in the table. The “Model performance” section provides values of performance indicators for test and training (placed in brackets) datasets for individual models

Among models based solely on tested signals (variants 1 to 4), the best results were obtained for τ_IR_*, captured using laser speckle imaging with an infrared laser. These models had a prediction error of 6% and high precision, with the highest number of observations classified as stress (Table 2, see Accuracy for variant 1). A comparably low prediction error of 8% was obtained for models built using the chlorophyll fluorescence signal. The detection accuracy of the remaining two signals did not exceed 90%, with the lowest result observed for τ_RED_*, which was also captured using a speckle-based method but using a red laser.

Relatively high accuracy was obtained for ΔRespiration, despite that this signal had the lowest correlation with stress (Table 1), of all predictors used for modelling. The ΔRespiration signal was calculated as the first derivative of CO_2_ respiration rate, therefore baseline shifts of the respiration signal were removed, which may be the reason for the better-than-expected performance.

Chlorophyll fluorescence is a well-established means of detecting anaerobic stress in apples [14, 17], with practical implementations in the form of advanced storage systems with dynamically controlled atmospheres [16]. However, in this study, the CF signal showed lower-than-expected accuracy. The lower predictive ability of the model could be explained by the variability in the amount of chlorophyll in the fruit. It should be noted that the fruit used in this study had a relatively long storage period (one to three months in cold storage). As the fruit matures, the chlorophyll in the fruit degrades [45, 46]. This causes the characteristic peak of the CF signal to be below the model's detection threshold, or to not appear at all, thus lowering the detection capability of the CF method. Such a situation was observed in the present study for the CF signal, while both τ_RED_*, τ_IR_* signals showed a characteristic increase in values. This may indicate a different physiological basis for the phenomena captured by laser speckle imaging.

For the τ_IR_*, τ_RED_* and CF-based models the addition of fruit mass resulted in an increase in prediction efficiency (variants 5, 6 and 7, see Table 2), reducing the detection error by 42% on average, compared with base models. Both the τ_RED_* and CF signals showed a high variance due to the differences in the pigment content of the fruit skin. The addition of indirect information about fruit ripeness, contained in the variable describing the mass of the apples, could allow the machine learning algorithm to compensate for the high variance of both signals. There is also a risk that this predictor caused overfitting of the model and its adaptation to local patterns specific to this dataset. Additional predictors (variants 9, 10 and 11, see Table 2) did not result in a significant increase in values of performance indicators for τ_IR_*, τ_RED_* and CF signals, showing that firmness and SSC did not bring new information to the model. Models based on the ΔRespiration coefficient with additional predictors (variants 9 and 12, see Table 2) did not result in an increase in values of performance indicators.

## Conclusions

The study showed that the signal from the scattered laser light phenomenon is a good predictor for the oxidative stress of apples. The results showed that effective prediction was possible using LSI and that no additional signals were required. Data obtained using the near-infrared laser (τ_IR_*) provided better prediction capabilities, compared to the laser with red light (τ_RED_*). Both CF and τ_IR_* signals were equally good for stress detection in principle. However, in an application with automatic detection based on machine learning models, the τ_IR_* signal proved to be superior, due to its stability and measurement repeatability. A comparison of different LSI signal processing methods showed that method based on the dynamics of changes in image content (τ_IR_* and τ_RED_*) were better indicators of stress than methods based on measurements of changes in pixel brightness (inertia moment or laser speckle contrast analysis). The proposed method has great application potential as a new predictor of fruit oxidative stress, which can be applied in modern storage systems with dynamically controlled atmospheres.

## Materials and methods

### Sample material

Apples of the ‘Najdared’ cultivar (*Malus x domestica *Borkh.) were used in this study. They were harvested within the optimum harvest window in 2022 and then stored in a cold room (normal atmosphere, 2 °C) for one month before testing. Tests in the climate chamber were carried out using fifteen apples. Before testing, the apples were taken out of cold storage and kept at room temperature for 10 h.

### Apple mass, volume and area

Each apple was weighed before the test in a climate chamber. Apple bulk volume was measured using the buoyancy method by submerging each apple in a bath of water and observing the increase in height of the water, corresponding to fruit volume. Each apple’s surface area was estimated to be the same as a sphere with the equivalent volume.

### Induced oxidative stress

The apples were exposed to a low-oxygen storage atmosphere to induce hypoxic stress. During each experimental run, the apples were kept in a small climate chamber—the Portable Gas-Exchange System GFS-3000 with Gas Exchange Chamber 3010 GWK1 (Heinz Walz GmbH, Effeltrich, Germany). Each test run consisted of pre-conditioning and storage of fruit in a normal ambient atmosphere at 22 °C for two hours. Then the air in the chamber was replaced by nitrogen to exclude oxygen for fruit respiratory processes. The apples were kept in this zero-oxygen storage atmosphere for 3.5 h, after which time the normal ambient storage atmosphere was restored for one hour. In total, a single run of the experimental procedure took 6.5 h. Measurements of fruit respiration rate and laser speckle imaging were carried out every eight minutes, and chlorophyll fluorescence was monitored every 60 s in parallel with changes in the storage atmosphere. The Oxygen Sensor with Amplifier-Box (Heinz Walz GmbH, Effeltrich, Germany) was used to monitor oxygen levels. With the gas flow rate set to 1250 μmol s^−1^, it took between eight and ten minutes for the oxygen content to decrease from ambient level to 0–0.02% in the fruit chamber. It took the same time to increase the O_2_ content back to ambient level.

### Fruit respiration rate

The rate of apples’ gas exchange was measured using Portable Gas-Exchange System GFS-3000 (Heinz Walz GmbH, Effeltrich, Germany) with modified Gas-Exchange Chamber 3010-GWK1 (Heinz Walz GmbH, Effeltrich, Germany). The modification involved placing a custom-made transparent plexiglass cover of 1480 ml volume on top of the chamber, enabling the whole apple to fit in the testing chamber. Gas exchange measurements based on actual CO_2_ levels were carried out every eight minutes. The apples’ surface temperature was measured with a thermocouple. Relative air humidity inside the climate chamber was set to 50% and the chamber temperature was maintained at 22 °C, the flow between the gas exchange system and the chamber was set at 1250 μmol s^−1^ and the gas inside the chamber was mixed with a built-in fan. The experiments were carried out in a dark room with no access to daylight.

### Chlorophyll fluorescence

Minimum chlorophyll fluorescence in the dark F_0_ was measured every 60 s using Imaging PAM Maxi (Heinz Walz GmbH, Effeltrich, Germany). The distance between the blue light (450 nm) LED array and the fruit surface was 25 cm, and the intensity of the measuring light was set at level 2. F_0_ was monitored in four different spots (c.a. 6–8 mm in diameter) on apple surfaces that were not irradiated with beams of both lasers. The average F_0_ from at least three sampling points was used for further analyses.

### Laser speckle imaging and analysis

**Fig. 4:**
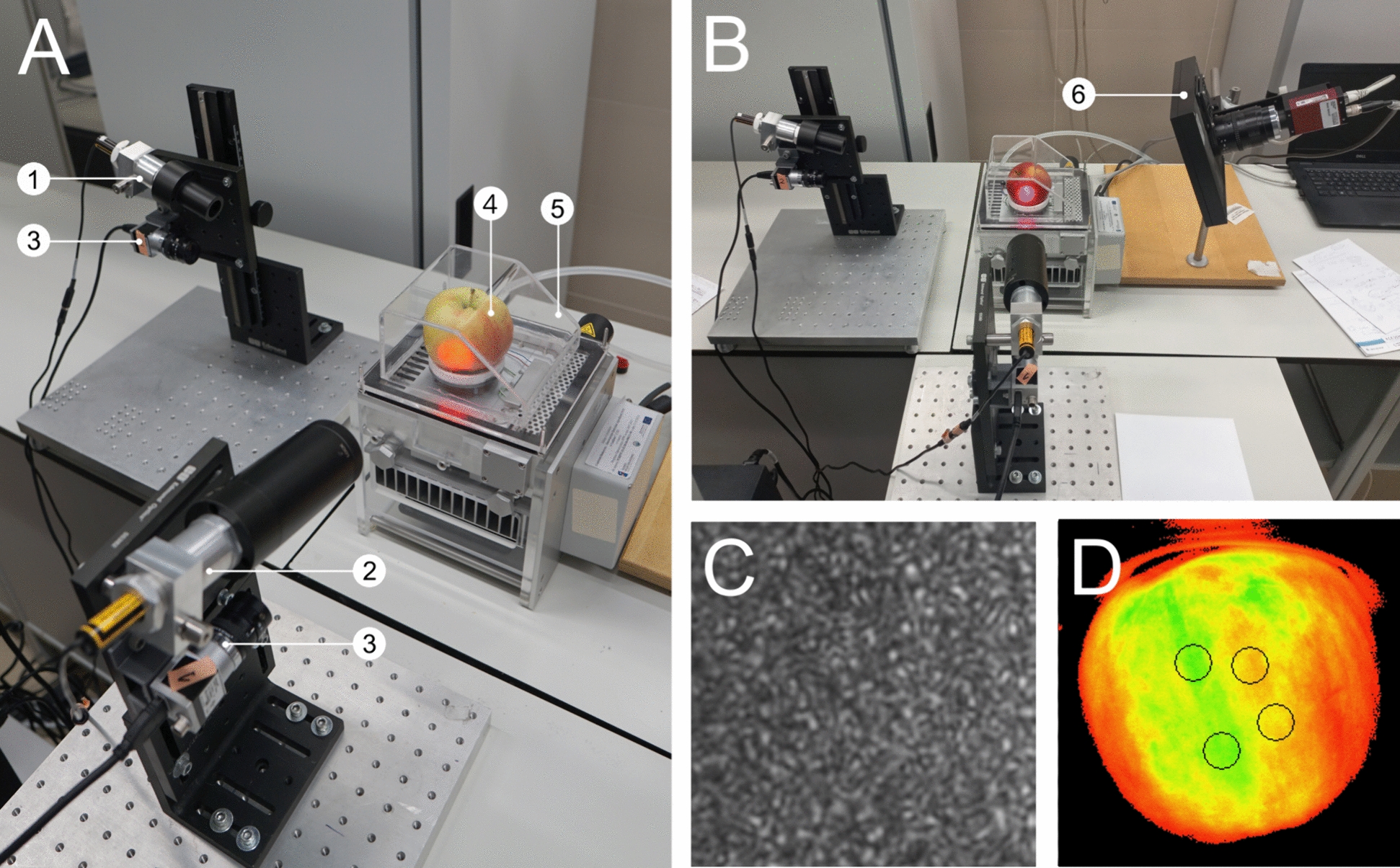
Laser speckle imaging system coupled with gas-exchange chamber and chlorophyll fluorescence measuring system (**A**, **B**) with indicated individual components (1—infrared laser with beam expander and removed focusing lens; 2—red laser with beam expander and focusing lens; 3—digital camera; 4—tested apple; 5—gas-exchange chamber; 6—camera and measuring light source of chlorophyll fluorescence recording system). Laser speckle patterns obtained from infrared laser (**C**). Map of sample chlorophyll fluorescence measuring points marked with circles (**D**)

Laser speckles were captured with a set of two Basler acA640-750 nm cameras (Basler AG, Ahrensburg, Germany), each equipped with a 25 mm TV lens 1:14 (Pentax Corporation, Tokyo, Japan). The lens aperture was fixed to f/16. Both cameras were placed ~ 25 cm from the sample's surface and set to cover a rectangular region of interest of dimensions equal to 128 by 128 pixels, which corresponded to a rectangular area of 8 mm by 8 mm. Videos of 60 s were captured at 30 frames per second every eight minutes. The optical axes of the cameras were perpendicular to each other and directed at the opposite sides of the fruit (Fig. 4a).

During each test in the climate chamber, the apples were constantly illuminated using 640 nm (RED) and 830 nm (IR) diode laser (640 nm, 40 MW of output power, MC6440O15-2A10, and 830 nm, 150 MW of output power, MC83150O15-2A10, both MONOCROM, Barcelona, Spain), directed perpendicular to the fruit. Both lasers were coupled with 10X Arcturus® HeNe beam expanders (Edmund Optics GmbH, Karlsruhe, Germany). The focusing lens was removed from the laser beam expander attached to the 830 nm laser to obtain a conical beam shape and evenly illuminate the entire captured area. Lasers were placed above the cameras with the angle of incidence of the beam equal to 15°. To obtain comparable pixel brightness for both cameras, the exposure time was set to 30,000 μs, with the gain set to 0.0 and 5.0 dB for the cameras capturing light from 640 and 830 nm lasers, respectively.

The rate of fluctuation of scattered laser light was estimated using a new coefficient describing the rate of decorrelation of speckle pattern from captured videos. To determine this coefficient, the correlation coefficients between the recorded video frames at different time intervals were first calculated for each video sequence. The obtained curve described the similarity between speckle patterns from the video sequences as a function of the time lag between them. Next, a stretched exponential model was fitted to the curve obtained in this procedure. The decorrelation rate was described using stretched exponential decay model, defined as:

$$CORR\left( t \right) = e^{{\left( {\frac{t}{\tau }} \right)^{\beta } }}$$

where CORR(t) is the correlation coefficient between two speckle patterns at time lag t; τ is the speckle pattern relaxation time in seconds; β is the scale coefficient. In further studies, the relaxation coefficient τ (tau) was used to detect the fruit’s oxidative stress. The higher the value of the coefficient τ, the slower the decorrelation rate of the speckle pattern.

Two previously developed coefficients of laser speckle fluctuations—temporary resolved laser speckle contrast (tLASCA) and inertia moment (IM)—were calculated in this study. The tLASCA was calculated as the ratio of standard deviation to the mean of the intensity for each pixle [47], calculated in the temporal domain (from all video frames) and averaged over the spatial domain (average from all pixels). It was expressed as:

$$tLASCA = \frac{1}{nm}\mathop \sum \limits_{x = m}^{1} \mathop \sum \limits_{y = n}^{1} \frac{{\sigma_{x,y,t} }}{{I_{x,y,t} }}$$

where *m* and *n* are the video frame dimensions, ${\sigma }_{x,y,t}$ and ${I}_{x,y,t}$ are the standard deviation and mean values of pixel intensities over all video frames at location *x*,*y*. Inertia Moment uses a grey-level co-occurrence matrix [47, 48], calculated from the time history of grey levels of all pixels from the sequence of speckle patterns [49]. Inertia moment was defined as:

$$I = \mathop \sum \limits_{i,j} GLCM\left[ {i,j} \right] \cdot \left( {i,j} \right)^{2}$$

where *GLCM* is the grey-level co-occurrence matrix and *i,j* are the row and column indexes of the *GLCM* matrix. Prior calculations of IM the GLCM was normalised using the total sum of all matrix entries. In the following parts of the article, subscript symbols RED and IR will refer to the laser speckle coefficients determined for 640 and 830 nm laser wavelengths, respectively.

### Firmness and soluble solid content

The firmness of individual apples was measured using a universal testing machine (Lloyd LRX, Lloyd Instruments Ltd., Hampshire, UK) with a 2500 N load cell. Apple skin was removed and the standard puncture test with a cylindrical probe (11.1 mm of diameter), at a crosshead speed set to 20 mm/min^−1^ and a maximum penetration depth equal to 8 mm was performed to determine firmness as the maximum value of force recorded during the test. For each apple, firmness was the average value from three measurements from the equatorial region of the fruit. The soluble solids content (SSC) was determined using a refractometer (PAL-BX/RI, ATAGO, Tokyo, Japan) by placing a drop of filtrate from a fresh apple onto a prism of the refractometer. The SSC content was expressed in %. The result for each apple was given as the average of three replicates.

### Data preparation and modelling

The aim of the machine learning algorithm was to classify each data point into two possible categories, namely ‘aerobic respiration’ or ‘hypoxic stress’. Therefore, stress detection was defined as a binary classification problem. Prediction models were built and validated using a Light Gradient Boosting Machine (LightGBM) algorithm [50]. This algorithm can be used for both classification and regression. LightGBM implements a boosting technique in which the learning process starts with a weak initial model, with new models trained iteratively, each adding to the prediction of the previous model to finally deliver a strong overall prediction [50]. In this study, the objective function for the LightGBM model was set to binary classification, and the evaluation metric was set to cross-entropy loss [51]. The boosting algorithm was set to ‘Gradient Boosting Decision Tree’ [52]. Tuning of the hyperparameters of the models was carried out during the preliminary tests, providing values for training rounds equal to 500, the maximum number of leaves in one tree equal to six and the learning rate equal to 0.05. Other parameters were set to default values in accordance with the relevant guidelines and regulations.

The algorithm implemented in this study belongs to the group of supervised learning algorithms. This means that, first, it was necessary to provide an exemplary classification of data made using the reference method. Model quality was assessed against the provided classification provided through expert examination of independent signals from three stress indicators—chlorophyll fluorescence, CO_2_ respiration rate and speckle pattern relaxation time τ. During the expert analysis, the data point was considered representative of hypoxic stress when at least two out of three signals showed considerable change in value levels when compared with levels recorded during storage in normal ambient atmosphere. The results of the expert analysis were used as ground truth for the machine learning algorithm. Prior analysis values of τ_RED,_ τ_IR_ were subjected to transformation using a natural logarithm. Thus, in the following sections of this paper, values of τ with an asterisk refer to their post-transformation form. Additionally, the first derivative of the CO_2_ respiration rate was used for classification purposes.

The complete set of predictor variables consisted of values of the relaxation coefficients (τ_RED_^*^ and τ_IR_^*^), minimum chlorophyll fluorescence (CF), CO_2_ respiration rate, mass, firmness and SSC for each apple. Modelling was carried out on a data set consisting of 605 observations. Model performance was estimated in a 20-fold cross-validation procedure with 70% of observations randomly selected for the train set and 30% for the test set. Twelve variants of models with different sets of predictor variables were tested. The choice of parameter combinations was motivated by the model’s utility value. Variants 1–4 involved τ_RED_*, τ_IR_*, CF or Δrespiration signals with no additional predictors. Variants 5–8 included mass of apple as additional and easy to measure predictor. Variants 5–8 allowed for non-destructive measurements. The following variants (9–12) involved destructive and time-consuming measurements and required an additional measuring apparatus.

Model performance was expressed using four metrics calculated from the confusion matrix on the test sets. Accuracy, precision, recall and F1 score were used to evaluate the performance of models. Accuracy is the proportion of correct predictions to the total number of predictions. Precision was the measure of data points correctly identified as indicating stress conditions out of all the data points identified as indicating stress by the model. Recall was the proportion of data points correctly identified by the model out of all actual data points indicating stress conditions. The F1 score was the harmonic mean of precision and recall. High values for F1 scores express a good balance between the precision and recall for the tested classifier.

The relative importance of variables in model predictions was estimated using gain. The gain implies the relative contribution of the corresponding feature to the model calculated by taking each feature’s contribution for each tree in the model. When compared to another feature, a higher value of this metric implies it is more important for generating a prediction. Gain was expressed as the relative ratio of the contribution of the selected variable to the overall predictive power of all parameters used in the model.

### Statistical analysis

Statistical tests (t-Student test) of the significance of differences between samples were performed using the ‘stats’ package (version 4.1.2.), which is a part of R (R Core Team, 2013).

## Supplementary Information

Supplementary Material 1.

## Data Availability

The datasets used and/or analysed during the current study are available from the corresponding author on reasonable request.
