# Supplementary material for: A novel application of laser speckle imaging technique for prediction of hypoxic stress of apples
Source: Plant Methods. 2024 Sep 28;20:147. doi: 10.1186/s13007-024-01271-7 (PMC11437772; doi:10.1186/s13007-024-01271-7)
Supplement: Supplementary file 1 — Supplementary Material 1. [file 13007_2024_1271_MOESM1_ESM.docx]

**A novel predictor for hypoxic stress of apples based on laser speckle imaging technique**

**Piotr Mariusz Pieczywek^[[1]](#footnote-1)^, Artur Nosalewicz, Artur Zdunek**

**Institute of Agrophysics, Polish Academy of Sciences, Doświadczalna 4, 20-290 Lublin, Poland**

**Table S1.** Results of measurements of basic physical parameters of individual apple fruit

| Sample | Mass  [g] | Firmess  [N] | SSC  [Brix] | Apple volume  [cm3] | Apple area  [cm2] |
| --- | --- | --- | --- | --- | --- |
| 1 | 173.90 | 40.16 | 12.46 | 194,4 | 162,3 |
| 2 | 171.40 | 40.68 | 11.46 | 190,4 | 160,1 |
| 3 | 164.80 | 40.34 | 13.62 | 172,0 | 149,6 |
| 4 | 168.50 | 38.69 | 11.85 | 188,0 | 158,7 |
| 5 | 163.60 | 40.85 | 13.05 | 181,6 | 155,1 |
| 6 | 166.70 | 38.78 | 13.66 | 177,0 | 152,4 |
| 7 | 202.40 | 39.82 | 11.70 | 240,0 | 186,8 |
| 8 | 157.40 | 40.84 | 13.50 | 175,2 | 151,4 |
| 9 | 156.10 | 45.61 | 12.43 | 173,6 | 150,5 |
| 10 | 160.80 | 50.50 | 14.87 | 182,4 | 155,5 |
| 11 | 163.60 | 45.19 | 12.33 | 185,0 | 157,0 |
| 12 | 162.60 | 40.43 | 12.36 | 180,8 | 154,6 |
| 13 | 154.00 | 40.20 | 11.83 | 170,4 | 148,6 |
| 14 | 147.50 | 37.44 | 12.96 | 164,0 | 144,9 |
| 15 | 145.50 | 37.72 | 12.26 | 166,4 | 146,3 |

1. Corresponding author: p.pieczywek@ipan.lublin.pl [↑](#footnote-ref-1)
